# Associations of food parenting practices in early childhood with dietary intake, BMI, and weight status in young adulthood: the KOALA Birth Cohort Study

**DOI:** 10.1186/s12937-026-01331-9

**Published:** 2026-05-12

**Authors:** Jelle Arts, Stef P. J. Kremers, Monique Mommers, Simone J. M. P. Eussen, Jessica S. Gubbels

**Affiliations:** 1 Department of Health Promotion, Maastricht University, NUTRIM Institute of Nutrition and Translational Research in Metabolism, PO Box 616, Maastricht, 6200 MD The Netherlands; 2 Department of Epidemiology, Care and Public Health Research Institute (CAPHRI), Maastricht University, PO Box 616, Maastricht, 6200 MD The Netherlands; 3 CARIM Cardiovascular Research Institute Maastricht, Maastricht University, PO Box 616, Maastricht, 6200 MD The Netherlands

## Abstract

**Background:**

Little is known about how early food parenting practices (FPPs) influence long-term diet and weight. This study examined associations of FPPs in early childhood— specifically restriction, pressure to eat, monitoring, and stimulation of healthy eating—with dietary intake, BMI, and weight status in young adulthood.

**Methods:**

We used data from the KOALA Birth Cohort Study in the Netherlands. At age 5 years, mothers reported FPPs using a 5-point Likert scale based on the Child Feeding Questionnaire (scale 1–5). At age 19 years, young adults self-reported their dietary intake and BMI via an online questionnaire, including a food frequency questionnaire. Food intake was categorized as 0–1, 2–3, or 4–7 days per week (scale 1–3). Associations were analyzed using ordinal logistic, binary logistic, linear, and robust regression models.

**Results:**

Questionnaires at both ages were available for 824 participants. The stimulation FPP “I make sure that my child eats enough healthy food products” was associated with lower unhealthy (B = -0.06), and higher healthy (B = 0.07) food intake. Regarding individual food categories, pressure to eat was associated with consuming more fried snacks (OR = 1.34), and less cooked vegetables (OR = 0.77). Restriction was associated with consuming more fried snacks (OR = 1.41), cookies (OR = 1.34), and pastries (OR = 1.42). Monitoring was associated with higher soda intake (OR = 1.29). The stimulation FPP was associated with consuming less fried snacks (OR = 0.67), cookies (OR = 0.70), and more fruits (OR = 1.65) and cooked vegetables (OR = 1.35). Regarding weight, pressure to eat was associated with lower BMI (B = -0.37) and reduced overweight risk (OR = 0.66), but these associations were nonsignificant after adjusting for BMI z-scores at age 5.

**Conclusions:**

Early FPPs may shape long-term dietary behaviors. Supportive, structured practices that stimulate healthy eating without coercion appear more beneficial than restrictive or pressuring practices. Longitudinal studies considering mediating and moderating factors are needed to clarify long-term associations between FPPs, dietary, and weight outcomes.

**Supplementary Information:**

The online version contains supplementary material available at 10.1186/s12937-026-01331-9.

## Introduction

Overweight is a major public health concern worldwide [1]. In 2022, 44% of adult women, and 43% of adult men were classified as overweight [1]. Overweight is a well-established risk factor for various health problems, including cardiovascular diseases, diabetes, and increased mortality risk [2, 3]. Furthermore, children who are overweight are more likely to become overweight adults, placing them at an early age at a heightened risk of developing non-communicable diseases [2, 3].

The development of overweight is primarily driven by dietary behaviors, resulting from an imbalance between energy intake and expenditure [4]. Early childhood, characterized by rapid growth and development, is a crucial period for establishing healthy dietary behaviors [5, 6]. During this period, parents play a pivotal role in shaping their children’s dietary behaviors as providers, role models, and regulators of their children’s food intake [5–7]. Therefore, food parenting practices (FPPs), defined as the strategies and behaviors parents use to influence their children’s eating habits, are particularly influential in early childhood [5, 6]. For example, parents can use specific practices to control children’s intake of healthy and unhealthy foods.

Commonly examined FPPs include restriction, pressure to eat, and monitoring [8]. Restriction involves enforcing strict limitations on a child’s access to foods or opportunities to consume them [9]. Pressure to eat refers to insisting, demanding, or physically struggling with a child in order to get the child to eat more food [9]. Monitoring entails keeping track of what and how much a child eats [9]. Several studies have investigated associations between these FPPs and children’s dietary intake [7, 10–13]. Three reviews, primarily based on cross-sectional studies, have reported inconsistent evidence regarding the associations of the FPPs monitoring, pressure to eat, and restriction in early childhood with children’s dietary behaviors [7, 11, 12]. However, these relationships are complex and often bidirectional [14, 15]. While FPPs can influence children’s eating behaviors, children’s own traits, such as food preferences, food intake, or weight status, may also influence how parents feed their children. For example, parents may use more restriction with children who are overweight or show strong preferences for unhealthy foods, or use more pressure to eat with children who are underweight or fussy eaters [16–18]. These dynamic relationships complicate the associations founds and may explain inconsistencies across studies.

Beside children’s dietary intake, FPPs have been associated to outcomes such as weight trajectories, appetite traits, and disordered eating behaviors throughout childhood and adolescence [8, 15, 19–22]. For example, food restriction has been associated with higher BMI and greater food responsiveness, whereas pressure to eat has been associated with lower BMI and reduced food enjoyment in childhood [21, 22]. During adolescence, these dynamics may contribute to the development of disordered eating behaviors [19, 20]. As dietary behaviors continue to develop from childhood through adolescence into young adulthood [23], parental influence also evolves. While parents largely control children’s food intake during early childhood, children gradually gain autonomy over their food choices and become increasingly influenced by peers and social norms [24, 25]. Given the dynamic relation between FPPs and dietary behaviors across developmental stages, it is important to longitudinally examine how early-life FPPs shape long-term dietary behaviors and weight outcomes [8].

While limited in number, longitudinal studies have more consistently suggested that children exposed to food restriction or pressure-to-eat are more likely to consume unhealthy foods [13, 26]. Interestingly, a recent retrospective study found that coercive controlling FPPs (including pressure to eat and restriction) during childhood were associated with more unhealthy food intake in adulthood [27]. Nonetheless, a systematic review of prospective studies found that restriction, pressure to eat, and monitoring were generally not associated with children’s weight outcomes [8]. However, higher-quality studies within this review suggested that pressure to eat might be associated with lower weight outcomes over time [8]. To date, studies examining the prospective associations between FPPs in early childhood and children’s dietary intake and weight outcomes have used follow-up periods ranging from 6 months to 6 years [8, 13]. Although such studies have provided valuable insights into the relatively short-term effects of FPPs on children’s dietary behaviors and weight outcomes, their longer-term impact across developmental phases remains largely unexplored.

In 2011, using data from the KOALA Birth Cohort Study, Gubbels et al. examined the FPPs monitoring, restriction, and stimulation of healthy intake (e.g., by getting child enthusiastic about healthy products). They found that both monitoring and stimulation of healthy intake were cross-sectionally associated with desirable dietary intake at age 5, while no association was found for restriction [28]. Stimulation of healthy intake was also significantly prospectively associated with lower BMI z-scores at age 7 [28]. Notably, the children from the KOALA Birth Cohort Study have now reached young adulthood, providing a unique opportunity to investigate how early-life FPPs may influence dietary intake and weight status later in life, with a 14-year follow-up. Understanding these long-term associations is important for informing early-life public health strategies aimed at preventing overweight and obesity in adulthood.

This study therefore aimed to examine the prospective associations between the FPPs restriction, pressure to eat, monitoring, and stimulation of healthy intake at approximately age 5 and dietary intake, BMI, and weight status at approximately age 19. By investigating these relationships, the study aims to provide valuable insights into the role of parents during early childhood in shaping long-term dietary behaviors and health outcomes.

## Methods

### Study design

We used data from two timepoints from the Koala Birth Cohort Study, a prospective cohort study in the Netherlands [29]. At the first timepoint children were aged approximately 5 years (range 3–6 years), and at the second timepoint they were young adults aged approximately 19 years (range 17–20 years). For consistency, these timepoints are hereafter referred to as age 5 and age 19. This study was reported in accordance with the STROBE guidelines for cohort studies (Additional file 1) [30].

### Participants and recruitment

Between 2000 and 2002, healthy pregnant women were included in the Koala Birth Cohort Study. Women were recruited from another cohort study on the etiology of pregnancy-related pelvic girdle pain (*n* = 2,375). Additionally, women were recruited through channels such as anthroposophist midwives, Steiner schools, organic food shops, and alternative lifestyle magazines (*n* = 502) [29]. The latter group was more likely to exhibit, at the time, alternative lifestyle practices, including nonstandard dietary habits (e.g., vegetarian or organic diets), parenting beliefs and methods and was called ‘alternative recruitment’ group. The former group was referred to as the ‘conventional recruitment’ group. In total, mothers of 2,877 children were included in the Koala Birth Cohort Study. All participants provided informed consent, and the study protocol was approved by the Maastricht University/University Hospital Maastricht medical ethics committee (MEC 01–139.3, 2001; MEC 00.182.15, 2006; and MEC 08-04-016, 2020).

Children included in the Koala Birth Cohort Study were eligible for inclusion in the current study, with children with congenital disorders (e.g., Down syndrome) or early childhood diseases (e.g., lung cancer) being excluded. Additionally, participants with incomplete questionnaires relevant to this study either at age 5 and/or 19 were excluded from analyses.

### Procedures

Mother-reported FPPs were collected between 2006 and 2008 when the child was aged 5 years old, using a hard copy questionnaire including the Child Feeding Questionnaire (CFQ) [31]. Between 2019 and 2021, the young adults’ self-reported dietary intake was assessed using an online questionnaire, including a food frequency questionnaire (FFQ), administered via the web-based platform Formdesk (Innovero Software Solutions BV).

### Parenting practices at age 5

The following mother-reported FPPs were assessed: pressure to eat, restriction, monitoring, and simulation of healthy eating. Pressure to eat, restriction, and monitoring were assessed through translated items of the CFQ, a self-report instrument developed by Birch et al. to assess parental beliefs, attitudes, and practices regarding child feeding [31]. Previous confirmatory factor analyses of the CFQ indicated acceptable factor loadings ranging from 0.37 to 0.95, and Cronbach’s alpha’s between 0.70 and 0.92 [31]. In the current study, pressure to eat, restriction, and monitoring were assessed using four, eight, and three items, respectively, with all Cronbach’s alpha values exceeding the threshold of 0.50 [32, 33]. Additionally, following Gubbels et al. we included two additional items on stimulation of healthy intake [28]: 1) “make sure that my child eats enough healthy food products”; and 2) “I get my child enthusiastic about healthy products, such as vegetables, fruit and wholegrain products”. These items have not been validated previously and were analyzed separately as single items, as they did not meet the Cronbach’s alpha threshold when combined [32, 33]. All statements were rated on a 5-point Likert scale. Monitoring items ranged from 1 (“never”) to 5 (“always”), while items for the other practices ranged from 1 (“completely disagree”) to 5 (“completely agree”). The specific items, response options, and corresponding Cronbach’s alpha values for each parenting practice are detailed in Additional file 2. Mean scores were calculated for the items corresponding to each parenting practice.

### Dietary intake at age 19

Dietary intake was assessed using an FFQ that was based on previous measurements in the Koala Birth Cohort Study [34]. This FFQ collected the weekly consumption of several food categories, of which we focused on seven unhealthy food categories, and four healthy food categories. Unhealthy food categories included: fried snacks; chips, nuts, or salty snacks; cake or cookies; pastries, chocolate bars or candy bars; candies; sugar-sweetened soda; and sugar-sweetened fruit juice/drinks. Healthy food categories included: fruits; salad and raw vegetables; cooked or stir-fried vegetables; and water. For each food category, consumption was assessed using one item per category, in which participants reported how often they consumed foods in that category during a typical week. The number of portions or portion sizes were not reported. The frequency of food intake was assessed using a six-point ordinal scale with the following response options: I do not consume this; less than one day per week; one day per week; 2–3 days per week; 4–5 days per week; and 6–7 days per week. To ensure sufficient data per category, these responses were recoded into three levels for analysis: 1) 0–1 days per week; 2) 2–3 days per week; and 3) 4–7 days per week. Additionally, based on these recoded values, separate mean scores were calculated for all unhealthy and all healthy food categories to assess overall dietary intake (range 1–3).

### Body mass index and weight status

At age 5, mothers reported their child’s height (cm) and weight (kg). In a subsample, trained research assistants measured the child’s height and weight during a home visit using a portable stadiometer (Leicester height measure) and a digital scale (CAS personal scale, HE-5), with values rounded to the nearest 0.1 unit [35]. Whenever available, data obtained during home visits were used; otherwise, the mother-reported data were used. The child’s height and weight were used to calculate BMI z-scores with the R packages anthro (version 1.0.1) for children younger than 60 months and anthroplus (version 1.0.0) for those older than 60 months, both developed by the World Health Organization [36, 37]. At age 19, the young adults self-reported their height and weight, from which BMI was calculated. Additionally, BMI of the young adults was categorized into weight status levels using binary indicators (yes/no) for underweight (< 18.5 kg/m²) and overweight (≥ 25 kg/ kg/m²).

### Covariates

Data were collected through questionnaires as part of the KOALA Birth Cohort Study, including the child’s sex (male or female), the dates of birth of both the child and the mother, parity (number of previous childbirths), maternal and paternal weight and height (used to calculate BMI), country of birth (recoded as Netherlands or other), and highest educational level (recoded as lower, medium, or higher based on the International Standard Classification of Education (ISCED) [38]). At age 19, data on the young adult’s living situation (recoded as living with parents or somewhere else) was collected through a questionnaire. Child age at both timepoints was calculated using the child’s date of birth and date of questionnaire completion. Similarly, maternal age at childbirth was calculated using the dates of birth of both the child and the mother. Moreover, information on recruitment group (conventional or alternative) was collected when providing consent for participation.

### Analyses

SPSS version 30 was used to explore the dataset and calculate descriptive statistics. RStudio version 2024.12.0 was employed for all subsequent analyses. Statistical significance was set at alpha = 0.05. First, we calculated means and standard deviations or numbers and percentages for the characteristics of participating parents and children at ages 5 and 19. One implausible BMI value at age 19 (53.2) was identified and excluded from the analysis. Additionally, we calculated means and standard deviations for all FPPs at age 5, and frequencies of dietary intake for all food categories at age 19. To explore the potential impact of participant drop-out between ages 5 and 19, we conducted independent-samples t-tests and chi-square tests on all FPP variables and participant characteristics, comparing the final sample with participants who provided data only at age 5.

Second, we examined prospective associations between parenting practices and both unhealthy and healthy food intake. FPPs (restriction, pressure to eat, monitoring, and the two stimulation items) were included as independent variables, while unhealthy or healthy food intake served as the dependent variable. Assumptions for linear regression were assessed: they were met for unhealthy food intake, but the assumption of normality was violated for healthy food intake. The assumption of normality remained unsatisfied after several transformations. Consequently, we performed a linear regression analysis for unhealthy food intake using the lm function in R, and a robust linear regression analysis for healthy food intake using the rlm function from the MASS package [39].

Third, we examined prospective associations between parenting practices and dietary intake of all individual food categories at follow-up, by conducting separate ordinal logistic regression analyses using the clm function from the ordinal package [40]. FPPs were included as independent variables, while dietary intake (per category) served as dependent variable. Assumptions for ordinal logistic regression, including the proportional odds (parallel lines) assumption, were checked and met. Odds ratios (ORs) were derived by exponentiating the estimated regression coefficients (B) from the ordinal logistic models. In these models B represents the estimated change in the log-odds of being in a higher intake level per one-unit increase in the FPP. The 95% confidence intervals (CIs) for the ORs were calculated by exponentiating the corresponding Wald confidence limits of B.

Fourth, associations between parenting practices and BMI were examined. Assumptions for linear regression were assessed, revealing a violation of the normality assumption. The assumption of normality remained unsatisfied after several transformations. Consequently, we conducted a robust linear regression analysis to examine associations between FPPs and BMI, using the rlm function from the MASS package [39].

Last, we examined the prospective associations between parenting practices and weight status (being underweight or overweight) using binary logistic regression analyses with the glm function. Again, FPPs were included as independent variables, while separate models were conducted for underweight and overweight as dependent variables. Assumptions for logistic regression were checked and met. For interpretability, ORs and CIs were calculated by exponentiating the estimated regression coefficients.

All analyses were conducted using complete case data. No imputation of missing values was performed. All models were adjusted for the child’s sex, age, and living situation, as well as the mother’s educational level, BMI, age, parity, and recruitment group. For sensitivity analysis, all models were also conducted with additional adjustment for the child’s BMI z-score at age 5. Additionally, to account for multiple testing, p-values for all models were adjusted using the Benjamini–Hochberg procedure [41], with non-significant associations after correction indicated in the results tables.

## Results

### Descriptives

**Fig. 1:**
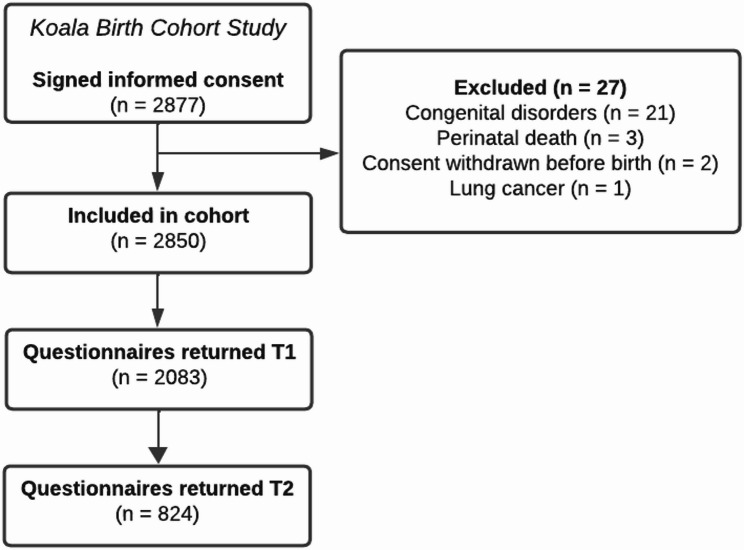
Participant flowchart at age 5 (T1) and age 19 (T2)

Among all included participants, 2,083 returned the questionnaire at age 5, and 884 at age 19. Completed questionnaires at both ages were available for 824 participants. Fig. 1 presents the participant flowchart. Table 1 provides an overview of the characteristics of participating children and their parents. Among participants with data available at both ages, age ranged from 3.84 to 6.84 years at the first timepoint and from 17.54 to 20.51 years at follow-up. Most children were aged 4 (59.0%) or 5 (36.3%), while most young adults were aged 18 (42.0%) or 19 (44.3%). The majority of mothers (62.5%) and fathers (61.4%) were highly educated, and most were born in the Netherlands (96.0% and 96.3%, respectively). Also, the majority of mothers had no (46.1%) or one (39.3%) previous born child. Additionally, a slight majority of the children were female (53.2%), and most lived with their parents at age 19 (80.1%). Furthermore, the majority of young adults were not considered underweight or overweight (76.8%), as reflected by a mean BMI of 22.04 ± 3.05. 

**Table 1:** Characteristics of participating parents and children at age 5 (T1) and age 19 (T2)

	*n* (%)	Mean (SD)
Highest educational level mother (*n* = 813)		
Higher	508 (62.5)	
Medium	269 (33.1)	
Lower	36 (4.4)	
Highest educational level father (*n* = 796)		
Higher	489 (61.4)	
Medium	232 (29.1)	
Lower	75 (9.4)	
Country of birth mother (*n* = 816)		
Netherlands	783 (96.0)	
Other	33 (4.0)	
Country of birth father (*n* = 816)		
Netherlands	786 (96.3)	
Other	30 (3.7)	
BMI mother at T1 (*n* = 813)		23.56 (3.51)
BMI father at T1 (*n* = 776)		24.70 (3.01)
Age mother at childbirth (years) (*n* = 824)		32.75 (3.67)
Recruitment group (*n* = 824)		
Conventional	627 (76.1)	
Alternative	197 (23.9)	
Parity (*n* = 820)		
0 previous births	378 (46.1)	
1 previous birth	322 (39.3)	
2 previous births	98 (12.0)	
3 previous births	20 (2.4)	
4 or more previous births	2 (0.2)	
Age child at T1 (years) (*n* = 824)		4.93 (0.53)
3 years old	13 (1.6)	
4 years old	486 (59.0)	
5 years old	316 (38.3)	
6 years old	9 (1.1)	
Sex child (*n* = 824)		
Male	386 (46.8)	
Female	438 (53.2)	
BMI z-score child at T1 (*n* = 781)		-0.38 (0.99)
Age child at T2 (years) (*n* = 824)		
17 years old	76 (9.2)	
18 years old	346 (42.0)	
19 years old	365 (44.3)	
20 years old	37 (4.5)	18.93 (0.68)
BMI child at T2 (*n* = 794)		22.04 (3.05)
Weight status at T2 (*n* = 794)		
Underweight	72 (9.1)	
Normal weight	610 (76.8)	
Overweight	112 (14.1)	
Living situation child at T2 (*n* = 824)		
With parent(s)	660 (80.1)	
Somewhere else	164 (19.9)	

Highest educational level was based on the International Standard Classification of Education (ISCED) [22]

Table 2 presents the mean scores of all FPPs at age 5, and Table 3 presents the dietary intake for all included food categories at age 19. On average, restriction was the least frequently used FPP (mean score = 3.32 ± 0.59), while stimulation of healthy intake was used most often (mean scores = 4.54 ± 0.71 and 4.68 ± 0.59 for the two stimulation items). The least frequently consumed food categories among young adults were fried snacks and sugar-sweetened fruit juice/drinks with 49.8% and 49.2%, respectively, consuming them less than twice per week. In contrast, water and cooked or stir-fried vegetables were consumed most frequently, with 89.2% and 78.4%, respectively, consuming them at least four times per week. 

**Table 2:** Mean score and standard deviation (SD) of each food parenting practice at age 5

Parenting practice	*N*	Mean (SD)
Pressure to eat	824	3.33 (0.75)
Restriction	824	3.32 (0.59)
Monitoring	823	4.41 (0.57)
Stimulation 1: I make sure that my child eats enough healthy food products	819	4.68 (0.59)
Stimulation 2: I get my child enthusiastic about healthy products, such as vegetables, fruit and wholegrain products	821	4.54 (0.71)

All parenting practices were assessed on a 5-point scale (1–5), using items from the Child Feeding Questionnaire (CFQ) [31] or Gubbels et al. [28]

**Table 3:** Dietary intake per food category at age 19 (*n* = 824)

Food category	0–1 day per week *n* (%)	2–3 days per week *n* (%)	4–7 days per week *n* (%)
Fried snacks	410 (49.8)	406 (49.3)	8 (1.0)
Chips, nuts, or savory snacks	143 (17.4)	593 (72.0)	88 (10.7)
Cake or cookies	226 (27.4)	401 (48.7)	197 (23.9)
Pastries, chocolate bars or candy bars	361 (43.8)	396 (48.1)	67 (8.1)
Candies	375 (45.5)	313 (38.0)	136 (16.5)
Fruits	90 (10.9)	261 (31.7)	473 (57.4)
Salad and raw vegetables	76 (9.2)	346 (42.0)	402 (48.8)
Cooked or stir-fried vegetables	14 (1.7)	164 (19.9)	646 (78.4)
Sugar-sweetened soda	348 (42.2)	310 (37.6)	166 (20.1)
Sugar-sweetened fruit juice/drinks	405 (49.2)	305 (37.0)	114 (13.8)
Water	22 (2.7)	67 (8.1)	735 (89.2)

The drop-out analysis showed that participants with data at both ages 5 and 19, compared to those with data only at age 5, scored significantly lower on pressure to eat (95% CI [-0.15, -0.00]), and higher on the stimulation FPP “I make sure that my child eats enough healthy food products” (MD = 0.07, 95% CI [0.02, 0.12]). No significant differences were found for restriction, monitoring, and the stimulation FPP “I get my child enthusiastic about healthy products, such as vegetables, fruit and whole meal products”. Participants with complete data were also significantly younger (MD = -0.11, 95% CI [-0.20, -0.02]), more often girls (χ² = 10.01, *p* = .002), and more frequently recruited from alternative lifestyle circles (χ² = 18.17, *p* < .001). Additionally, both parents had higher educational levels (mothers: χ² = 50.03, *p* < .001; fathers: χ² = 43.63, *p* < .001) and lower BMI values (mothers: MD = -0.62, 95% CI [-0.96, -0.30]; fathers: MD = -0.55, 95% CI [-0.84, -0.26]). Moreover, mothers were significantly older (MD = 0.74, 95% CI [0.41, 1.07]). No significant differences were found for parity or parents’ country of birth.

### Associations of food parenting practices with dietary intake, BMI, and weight status

Table 4 presents the prospective associations between FPPs and dietary intake, BMI, and weight status. Table 2 in Additional file 3 presents the same findings, but with additional adjustment for BMI z-scores at age 5. Regarding overall dietary intake, we found a significant association between the stimulation FPP “I make sure that my child eats enough healthy food products” at age 5 with lower consumption of unhealthy food (B = -0.06, 95% CI [-0.10, -0.02]), and higher consumption of healthy food (B = 0.07, 95% CI [0.03, 0.11]) at age 19. After adjusting for BMI z-scores, we found a significant association between the stimulation FPP “I get my child enthusiastic about healthy products, such as vegetables, fruit and wholegrain products” at age 5 and a higher intake of healthy food at age 19 (B = 0.03, 95% CI [0.00, 0.07]).

**Table 4:** Prospective associations between food parenting practices at age 5 and food intake (healthy/unhealthy and by category; *n* = 792), BMI, underweight, and overweight at age 19 (*n* = 765)

Outcome	Pressure to eat	Restriction	Monitoring	Stimulation 1^a^	Stimulation 2^b^
	B or OR^c^ [95% CI]	B or OR^c^ [95% CI]	B or OR^c^ [95% CI]	B or OR^c^ [95% CI]	B or OR^c^ [95% CI]
Unhealthy food	0.03 [-0.00, 0.06]	0.03 [-0.01, 0.07]	0.03 [-0.01, 0.07]	**-0.06 [-0.10**,** -0.02]^**	-0.02 [-0.05, 0.01]
Fried snacks	**1.34 [1.08**,** 1.65]^**	**1.41 [1.08**,** 1.83]^**	1.08 [0.82, 1.41]	**0.67 [0.50**,** 0.88]**	0.87 [0.70, 1.08]
Chips, nuts, or savory snacks	1.15 [0.92, 1.43]	1.02 [0.78, 1.34]	1.10 [0.83, 1.45]	0.97 [0.73, 1.29]	0.84 [0.66, 1.06]
Cake or cookies	1.14 [0.94, 1.37]	**1.34 [1.06**,** 1.69]**	0.94 [0.74, 1.19]	**0.70 [0.55**,** 0.89]**	1.05 [0.87, 1.27]
Pastries, chocolate bars or candy bars	0.95 [0.78, 1.15]	**1.42 [1.11**,** 1.81]**	1.06 [0.82, 1.36]	0.78 [0.61, 1.00]	0.96 [0.79, 1.17]
Candies	1.13 [0.94, 1.37]	0.94 [0.74, 1.18]	1.04 [0.81, 1.33]	0.88 [0.69, 1.12]	0.98 [0.81, 1.20]
Sugar-sweetened soda	1.10 [0.91, 1.33]	0.87 [0.69, 1.10]	**1.29 [1.01**,** 1.65]**	0.87 [0.67, 1.11]	0.91 [0.75, 1.11]
Sugar-sweetened fruit juice/drinks	1.02 [0.84, 1.23]	0.98 [0.77, 1.25]	1.16 [0.90, 1.48]	1.07 [0.84, 1.37]	0.89 [0.74, 1.08]
Healthy food	-0.01 [-0.04, 0.02]	-0.02 [-0.05, 0.02]	-0.03 [-0.07, 0.01]	**0.07 [0.03**,** 0.11]**	0.03 [0.00, 0.07]
Fruits	1.04 [0.85, 1.27]	1.11 [0.86, 1.42]	0.94 [0.73, 1.22]	**1.65 [1.28**,** 2.13]**	1.07 [0.86, 1.32]
Salad and raw vegetables	1.04 [0.85, 1.26]	0.83 [0.65, 1.06]	0.83 [0.64, 1.06]	1.12 [0.87, 1.43]	1.18 [0.96, 1.45]
Cooked or stir-fried vegetables	**0.77 [0.60**,** 1.00]^**	0.94 [0.69, 1.28]	0.84 [0.61, 1.15]	**1.35 [1.00**,** 1.81]^**	1.21 [0.95, 1.54]
Water	0.72 [0.51, 1.02]	0.96 [0.65, 1.44]	0.72 [0.47, 1.13]	1.02 [0.68, 1.55]	1.00 [0.71, 1.41]
BMI	**-0.37 [-0.65**,** -0.11]^**	0.07 [-0.27, 0.40]	0.08 [-0.27, 0.43]	0.30 [-0.05, 0.65]	0.10 [-0.19, 0.38]
Underweight	1.40 [0.97, 2.04]	1.27 [0.80, 2.00]	0.97 [0.62, 1.52]	0.80 [0.51, 1.24]	0.83 [0.59, 1.17]
Overweight	**0.66 [0.49**,** 0.90]^**	1.27 [0.87, 1.86]	1.12 [0.75, 1.68]	1.30 [0.85, 2.00]	1.09 [0.78, 1.52]

All food parenting practices were assessed on a 5-point scale (1–5), using items from the Child Feeding Questionnaire (CFQ) [31] or adapted from Gubbels et al. [28]

All models were adjusted for the child’s sex, age, living situation, the mother’s educational level, age, BMI, parity, and recruitment group

Significant (*p* < .05) associations are indicated in bold font

^a^“I make sure that my child eats enough healthy food products”

^b^“I get my child enthusiastic about healthy products, such as vegetables, fruit and wholegrain products”

^c^BMI, as well as mean healthy and unhealthy food intake (each ranging from 1 to 3), were treated as continuous variables. For these outcomes, B values were calculated. Individual food categories were treated as ordinal variables with three levels (1–3), while underweight and overweight status were treated as binary outcomes (yes/no). For the ordinal and binary outcomes, odds ratios (ORs) were calculated from the estimated regression coefficients

^^^Indicates associations no longer significant after correcting for multiple testing using the Benjamini–Hochberg procedure [41]

Regarding individual food categories, we found that pressure to eat at age 5 was significantly associated with poorer dietary intake at age 19, including higher odds of consuming fried snacks (OR = 1.34, 95% CI [1.08, 1.65]), and lower odds of consuming cooked or stir-fried vegetables (OR = 0.77, 95% CI [0.60, 1.00]). Similarly, restriction at age 5 was significantly associated with higher odds of consuming fried snacks (OR = 1.41, 95% CI [1.08, 1.83]), cake or cookies (OR = 1.34, 95% CI [1.06, 1.69]), and pastries, chocolate bars, or candy bars (OR = 1.42, 95% CI [1.11, 1.81]) at age 19. In addition, monitoring at age 5 was significantly associated with higher odds of consuming sugar-sweetened soda (OR = 1.29, 95% CI [1.01, 1.65]) at age 19. In contrast, the stimulation FPP “I make sure that my child eats enough healthy food products” at age 5 was associated with healthier dietary intake at age 19, including lower odds of consuming fried snacks (OR = 0.67, 95% CI [0.50, 0.88]), cake or cookies (OR = 0.70, 95% CI [0.55, 0.89]), as well as higher odds of consuming fruits (OR = 1.65, 95% CI [1.28, 2.13]), and cooked or stir-fried vegetables at age 19 (OR = 1.35, 95% CI [1.00, 1.81]). We found no significant association between the stimulation FPP “I get my child enthusiastic about healthy products, such as vegetables, fruit, and wholegrain products” and dietary intake of individual food categories. After additional adjustment for BMI z-scores at age 5, the majority of associations remained unchanged. Only the association between pressure to eat and intake of cooked or stir-fried vegetables was no longer significant (see Additional file 3, Table 2).

Regarding BMI and weight status, in models without adjustment for BMI z-scores at age 5, pressure to eat at age 5 was significantly associated with lower BMI at age 19 (B = -0.37, 95% CI [-0.65, -0.11]). Similarly, pressure to eat was associated with lower odds of overweight at follow-up (OR = 0.66, 95% CI [0.49, 0.90]). However, these associations were no longer significant after adjusting for BMI z-scores at age 5. For the other FPPs, no significant associations were found with BMI, underweight, or overweight.

## Discussion

This study investigated the prospective associations between several FPPs assessed at age 5 and dietary intake and weight assessed at age 19. We found that the FPPs pressure to eat, restriction, and monitoring were each significantly associated with less healthy dietary intake or more unhealthy dietary intake of at least one food category at follow-up 14 years later. Regarding stimulation of healthy intake, we found that one of the stimulating practices was associated with healthier dietary intake of several food categories in young adulthood, while we found no significant association between another stimulating practice and dietary intake. Additionally, only pressure to eat was significantly associated with lower BMI and lower odds of overweight at follow-up. However, these associations were no longer found when analyses were adjusted for BMI z-scores at age 5.

In the current study, food restriction was associated with more consumption of unhealthy foods (i.e., fried snacks; cake or cookies; and pastries, chocolate bars or candy bars) in the long term. This finding contrasts with the cross-sectional results of Gubbels et al. (2011), who reported no association between restrictive feeding practices and dietary intake [28]. It is possible that the non-beneficial effects of restrictive parenting practices emerge over time, a notion supported by most short-term prospective studies, which have found associations between restriction and increased intake of unhealthy foods [13]. Additionally, consistent with the majority of previous research on the relatively short-term effects of FPPs [7], pressure to eat was associated with less consumption of healthy food (i.e., cooked or stir-fried vegetables) and more consumption of unhealthy food (i.e., fried snacks) in the long term. Pressure to eat and food restriction are often seen as coercive controlling practices, which refers to pushing and dominating the child to behave according to parents’ desires [8, 9, 42]. In line with our findings, a recent retrospective study found that less coercive controlling FPPs during childhood were associated with healthier food intake in adulthood, including lower consumption of savory and sweet foods [27]. Together, these findings support the suggestion that coercive controlling FPPs in early childhood may disrupt the development of intrinsic self-regulation of eating, while developing aversions or desires for certain foods [9, 19, 27]. For example, pressure to eat could develop aversions to the foods being pressured. Additionally, restriction of foods could make these foods more desirable for the child, resulting in overconsumption when available [19, 27]. These mechanisms would explain the long-term associations of coercive controlling practices with poorer dietary intake found in the current study. However, it should be noted that in the sensitivity analyses, where we adjusted for children’s BMI z-scores at age 5, the association between pressure to eat and lower consumption of cooked or stir-fried vegetables was no longer significant. These findings may be explained by parents tending to apply more pressure to eat when they are concerned about their children being underweight [17, 43]. Together, these findings highlight the complex and potentially bidirectional relationships between parental feeding practices, dietary behaviors, and weight outcomes [14, 15]. Nevertheless, also with adjusting for BMI-z scores, the majority of associations with dietary intake remained. Although the long-term associations of pressure to eat and food restriction with poorer dietary intake may not imply causality, our findings suggest that such parenting practices in early childhood could negatively influence dietary behaviors into adulthood.

For monitoring, we found a significant association only with higher intake of sugar-sweetened soda. In contrast, Gubbels et al. [28] previously found that monitoring was cross-sectionally associated with desirable dietary intake at age 5. Previous research has generally also reported inconsistent associations between parental monitoring and children’s dietary intake [9, 12]. However, two longitudinal studies involving 4-year-olds, with follow-up periods of 3 and 6 years, reported significant associations between monitoring and reduced consumption of sugar-rich foods and beverages [44, 45]. Monitoring is commonly classified under structured parenting practices, which involve organizing the child’s environment to support healthy eating behaviors [9]. While some studies suggest that structured practices like monitoring can positively influence children’s future dietary habits [13], our findings do not support this effect over the long term. Further longitudinal research is needed to explore the lasting impact of parental monitoring on children’s dietary behavior.

As regards stimulation of healthy intake, parents making sure that their child ate enough healthy foods was positively associated with healthier dietary intake at follow-up, consistent with the previous cross-sectional findings from Gubbels et al. These findings support the idea that structured and supportive approaches to healthy eating without coercion may promote more favorable long-term dietary habits. In contrast, getting children enthusiastic about healthy products was not significantly associated with dietary intake in young adulthood. However, when adjusting for BMI z-scores at age 5, we found an association between this practice and healthy food intake. This could perhaps be explained by parents’ increased use of this practice when they are concerned about their child’s weight (i.e., a higher BMI z-score). This interpretation is supported by the previous cross-sectional findings of Gubbels et al., who found a positive association between BMI z-scores at age 5 and stimulation of healthy eating [28]. Although autonomy-supportive FPPs such as encouragement have received less attention in existing research, previous studies have generally found positive associations between encouragement and children’s fruit and vegetable intake [9]. However, our findings did not consistently confirm a long-term association with dietary intake in young adulthood. This could be perhaps explained by the limitations of assessing this practice using single items. As previously suggested, understanding the impact of encouragement-based practices necessitates a more deliberate conceptualization and comprehensive assessment [9]. Therefore, we recommend that future studies examining FPPs related to encouragement, as well as their association with dietary intake, use validated instruments, such as the more recently developed HomeSTEAD Family Food Practices Survey [46]. Subsequently, we encourage future studies to further examine the long-term effects of structure- and autonomy-supportive FPPs on diet and weight outcomes.

Regarding BMI and weight status, we found no significant prospective associations with any FPPs after adjusting for BMI z-scores at age 5. Without this adjustment, only pressure to eat was significantly associated with lower BMI and lower odds of having overweight at follow-up. This association is also likely driven by reverse causality: parents may apply more pressure to eat in response to their children having a relatively lower weight, rather than this practice causing lower weight [14, 17, 47]. Accordingly, BMI at age 5 may act as a mediator in the relationship between pressure to eat and later weight outcomes. While some previous prospective studies also found that pressure to eat was associated with lower weight, most have generally shown that the FPPs restriction, pressure to eat, and monitoring were not consistently associated with changes in children’s weight status over time [8]. Additionally, Gubbels et al., only found a significant short term prospective association between stimulation of healthy intake and lower BMI, but not with the FPPs monitoring and restriction [28]. Our findings, along with those of previous studies, suggest that while FPPs in early childhood may shape dietary intake throughout childhood and at least up to young adulthood, their impact on BMI and weight status is more complex as these are influenced by a wider range of interacting factors, including physical activity, sedentary behavior, and environmental factors [48–50]. Thus, dietary behaviors and weight are related but distinct outcomes, influenced by partially overlapping but not identical determinants [51–53]. Therefore, we recommend that future studies examining the long-term effects of FPPs on BMI and/or weight status also take related energy balance-related behaviors, such as physical activity, into account.

It should be noted that after correcting for multiple testing, some associations were no longer statistically significant. Nevertheless, restriction remained significantly associated with increased intake of unhealthy foods (i.e., cake or cookies; and pastries, chocolate bars, or candy bars) after adjustment for multiple testing. Additionally, the stimulation FPP remained significantly associated with lower intake of unhealthy foods (i.e., fried snacks; and cakes or cookies) and higher intake of healthy foods (i.e., fruits).

Given the long-term associations between FPPs and dietary intake identified in the current study, public health strategies are recommended to promote structured, supportive, and non-coercive approaches to feeding. Public health professionals are encouraged to guide parents in avoiding the use of pressure or restriction. Supporting parents in adopting more structured and supportive feeding strategies may help foster healthier dietary intake across development, taking into account the complex, dynamic and bidirectional relationships between parenting practices and children’s dietary behaviors and weight [14].

### Strengths and limitations

The current study had several strengths and limitations. A key strength is the 14-year longitudinal prospective follow-up, which allowed for the examination of long-term effects of early childhood FPPs, substantially longer than any previous study on this topic (maximum 6 years follow-up [8, 13]). Another strength is the large sample size of children with complete questionnaire data at both ages (*n* = 824). Moreover, the use of validated scales from the CFQ to assess the FPPs monitoring, pressure to eat, and restriction is a strength [31].

A limitation of the current study is that all data, including the FPPs, dietary intake, and BMI, were self-reported which may have introduced social desirability or recall bias [54, 55]. For example, parents may have underreported less desirable FPPs such as pressure to eat, while young adults may have underreported their intake of unhealthy foods [54]. Such bias could have led to an underestimation of the true associations between FPPs and dietary behaviors. Additionally, the questionnaire at age 19 was self-reported by the young adults, whereas at age 5 it was completed by their parents. This discrepancy may have influenced the outcomes and complicates comparisons with earlier findings. Furthermore, we only assessed food frequency intake per category in terms of days per week, which does not capture more specific quantitative information such as the number of portions or portion sizes. Moreover, the drop-out of participants between ages 5 and 19 may have resulted in selection bias. This concern is supported by our drop-out analysis, which showed significant differences on two FPPs and several sociodemographic characteristics between the final sample and those who only participated at age 5. Such differences further complicate direct comparisons over time. Another limitation is the relatively low Cronbach’s alpha values of the food parenting scales. Although values above .50 are sometimes considered acceptable in research [33], higher thresholds (e.g., above .60 or .70) are generally preferred for internal consistency [32]. Therefore, particularly for the pressure to eat scale (α = .50), these results should be interpreted with caution. Besides, as a result of not meeting the Cronbach’s alpha threshold, the two items corresponding to stimulation of healthy intake were analyzed separately. Also, since the stimulation of healthy intake has been specifically studied only by Gubbels et al., our ability to compare the findings for this practice with those of other previous studies and populations was limited. Another limitation is the overrepresentation of parents recruited from ‘alternative lifestyle’ circles [29]. As a result, caution is needed when generalizing our findings to the broader Dutch population. However, all analyses were adjusted for recruitment group. Furthermore, FPPs were assessed at a single timepoint, which may not capture changes in or stability of practices across development. In addition, some potentially important nutritional foods, including protein sources, were not examined, which can be considered a limitation. Future studies using more comprehensive dietary data are needed to examine these food categories in greater detail. Moreover, there may be confounding, mediating, or moderating factors that were not accounted for in our analysis, which could have influenced the associations found. These factors could include confounders such as young adults’ educational level [56], potential mediators such as children’s food intake at age 5 [23, 57, 58], and moderators such as parenting style, children’s age, or appetitive traits [59–61]. Future studies are recommended to account for additional confounding factors, and examine potential mediating or moderating factors, as well as bidirectionality of associations, to better understand the complex relationships between FPPs, dietary behaviors, and weight outcomes.

## Conclusions

We found several associations between FPPs in early childhood and food intake in young adulthood. Overall, pressure to eat and food restriction were associated with less favorable dietary intake, whereas a stimulating practice was associated with more favorable dietary intake. Although pressure to eat was initially associated with lower weight at follow-up, this association did not remain after adjusting for early BMI z-scores, indicating a complex and potentially bidirectional relationship. Strategies to promote lifelong healthy dietary behaviors are recommended to focus on structured and supportive, non-coercive parenting approaches in early childhood to ensure the intake of healthy foods, rather than pressuring or restricting children’s eating. Future longitudinal studies considering confounding, mediating, and moderating factors are needed to better understand the long-term associations of FPPs on dietary and weight outcomes.

## Supplementary Information

Supplementary Material 1.

Supplementary Material 2.

Supplementary Material 3.

## Data Availability

Data that support the findings of the study are available from the corresponding author upon reasonable request.

## Abbreviations

BMI: Body max index
CFQ: Child Feeding Questionnaire
FFQ: Food frequency questionnaire
FPPs: Food parenting practices
