# Supplementary material for: Associations of food parenting practices in early childhood with dietary intake, BMI, and weight status in young adulthood: the KOALA Birth Cohort Study
Source: Nutr J. 2026 May 12;25:72. doi: 10.1186/s12937-026-01331-9 (PMC13335302; doi:10.1186/s12937-026-01331-9)
Supplement: Supplementary file 2 — Supplementary Material 2. [file 12937_2026_1331_MOESM2_ESM.pdf]

Additional file 2.

Table 1. Items, response options, and internal consistency coefficients of the assessed food parenting practices.

Practice	items	Response options	Chronbach's α
**Pressure to eat**	1. My child should always eat all of the food on his/her plate<br>2. I have to be especially careful to make sure my child eats enough<br>3. If my child says ''I'm not hungry'', I try to get him/her to eat anyway<br>4. If I did not guide or regulate my child's eating, (s)he would eat much less than she should	A	.50
**Restriction**	1. I have to be sure that my child does not eat too many sweets (candy, ice cream, pastries).<br>2. I have to be sure that my child does not eat too many high-fat foods.<br>3. I have to be sure that my child does not eat too much of his/her favorite foods.<br>4. As a reward for good behavior, I offer my child his/her favorite foods.<br>5. As a reward for good behavior, I offer my child sweets (candy, ice cream, pastries)<br>6. If I did not guide or regulate my child's eating, (s)he would eat too much of his/her favorite foods.<br>7. If I did not guide or regulate my child's eating, (s)he would eat too many junk foods.<br>8. I intentionally keep some foods out of my child's reach.	A	.61
**Monitoring**	1. How much do you keep track of the sweets (candy, ice cream, pies, pastries) that your child eats?<br>2. How much do you keep track of the snack food (potato chips, nuts, cheese puffs) that your child eats?<br>3. How much do you keep track of the high-fat foods that your child eats	B	.85
**Stimulation 1**	I make sure that my child eats enough healthy food products	A	-
**Strimulation 2**	I get my child enthusiastic about healthy products, such as vegetables, fruit and whole meal products	A	-

A indicates response options: 1) completely disagree; 2) slightly disagree; 3) neutral; 4) slightly agree; 5) completely agree

B indicates response options: 1) never; 2) rarely; 3) sometimes; 4) mostly; 5) always
