# Supplementary material for: Associations of food parenting practices in early childhood with dietary intake, BMI, and weight status in young adulthood: the KOALA Birth Cohort Study
Source: Nutr J. 2026 May 12;25:72. doi: 10.1186/s12937-026-01331-9 (PMC13335302; doi:10.1186/s12937-026-01331-9)
Supplement: Supplementary file 3 — Supplementary Material 3. [file 12937_2026_1331_MOESM3_ESM.pdf]

# Additional file 3.

**Table 1.** Results of the drop-out analysis comparing the final sample with the drop-out sample.

| Variable | Final sample * | Drop-out sample ** | p-value |
|---|---|---|---|
| Participant characteristics | | | |
| Highest educational level mother (Higher/Medium/Lower) | **508/269/36** | **593/503/132** | **<.001** |
| Highest educational level partner (Higher/Medium/Lower) | **489/232/75** | **560/451/187** | **<.001** |
| Country of birth mother (Netherlands/Other) | 783/33 | 1207/32 | .064 |
| Country of birth father (Netherlands/Other) | 786/30 | 1181/47 | .861 |
| BMI mother (mean and SD) | **23.56 (3.52)** | **24.20 (3.81)** | **<.001** |
| BMI father (mean and SD) | **24.71 (3.02)** | **25.27 (3.26)** | **<.001** |
| Age mother in years (mean and SD) | **32.75 (3.67)** | **32.02 (3.79)** | **<.001** |
| Parity (mean and SD) | 0.72 (0.80) | 0.69 (0.72) | .424 |
| Recruitment group (Conventional/Alternative) | **627/197** | **1053/206** | **<.001** |
| Age child in years (mean and SD) | **4.92 (0.59)** | **5.03 (1.21)** | **.017** |
| Sex child (Male/Female) | **386/438** | **679/580** | **.002** |
| Food parenting practices | | | |
| Pressure to eat (mean and SD) | **3.46 (0.82)** | **3.54 (0.64)** | **.037** |
| Restriction (mean and SD) | 3.32 (0.59) | 3.31 (0.62) | .788 |
| Monitoring (mean and SD) | 4.41 (0.57) | 4.41 (0.56) | .957 |
| Stimulation 1: I make sure that my child eats enough healthy food products (mean and SD) | **4.68 (0.59)** | **4.61 (0.61)** | **.010** |
| Stimulation 2: I get my child enthusiastic about healthy products, such as vegetables, fruit and wholegrain products (mean and SD) | 4.54 (0.71) | 4.51 (0.74) | .280 |

* Participant with complete questionnaire data at both ages 5 and 19

** Participants with questionnaire data only at age 5

Note: Significant differences are indicated in **bold font**

**Table 2.** Prospective associations between food parenting practices at age 5 and food intake (healthy/unhealthy and by category; n = 756), BMI, underweight, and overweight at age 19 (n = 732), adjusted for BMI z-scores at age 5.

| Outcome | Pressure to eat | Restriction | Monitoring | Stimulation 1[a] | Stimulation 2[b] |
|---|---|---|---|---|---|
| | **B or OR[c] (95% CI)** | **B or OR[c] (95% CI)** | **B or OR[c] (95% CI)** | **B or OR[c] (95% CI)** | **B or OR[c] (95% CI)** |
| Unhealthy food | 0.03 [-0.01, 0.08] | 0.04 [-0.00, 0.08] | 0.02 [-0.01, 0.06] | **-0.06 [-0.10, -0.01]^** | -0.02 [-0.06, 0.01] |
| Fried snacks | **1.28 [1.03, 1.60]^** | **1.49 [1.14, 1.95]^** | 1.16 [0.88, 1.53] | **0.68 [0.51, 0.91]^** | 0.80 [0.64, 1.01] |
| Chips, nuts, or savory snacks | 1.13 [0.90, 1.42] | 1.04 [0.79, 1.37] | 1.13 [0.84, 1.50] | 1.04 [0.77, 1.40] | 0.84 [0.66, 1.08] |
| Cake or cookies | 1.10 [0.91, 1.34] | **1.41 [1.11, 1.79]** | 0.97 [0.76, 1.25] | **0.67 [0.52, 0.86]** | 1.08 [0.89, 1.33] |
| Pastries, chocolate bars or candy bars | 0.95 [0.78, 1.16] | **1.47 [1.14, 1.88]** | 1.04 [0.80, 1.34] | 0.84 [0.65, 1.09] | 0.92 [0.74, 1.13] |
| Candies | 1.13 [0.93, 1.37] | 0.96 [0.75, 1.21] | 1.05 [0.82, 1.35] | 0.85 [0.66, 1.09] | 0.98 [0.80, 1.20] |
| Sugar-sweetened soda | 1.02 [0.84, 1.24] | 0.90 [0.71, 1.14] | **1.35 [1.05, 1.74]^** | 0.86 [0.66, 1.11] | 0.90 [0.74, 1.11] |
| Sugar-sweetened fruit juice/drinks | 0.97 [0.80, 1.19] | 0.97 [0.76, 1.24] | 1.17 [0.91, 1.51] | 1.04 [0.81, 1.34] | 0.92 [0.75, 1.13] |
| Healthy food | -0.03 [-0.07, 0.01] | -0.02 [-0.05, 0.02] | -0.01 [-0.04, 0.02] | **0.07 [0.03, 0.11]** | **0.03 [0.00, 0.07]** |
| Fruits | 1.08 [0.87, 1.33] | 1.10 [0.86, 1.43] | 0.92 [0.71, 1.20] | **1.68 [1.29, 2.20]** | 1.08 [0.87, 1.35] |
| Salad and raw vegetables | 1.05 [0.85, 1.28] | 0.85 [0.67, 1.10] | 0.87 [0.67, 1.13] | 1.09 [0.84, 1.42] | 1.20 [0.97, 1.48] |
| Cooked or stir-fried vegetables | 0.77 [0.59, 1.01] | 0.96 [0.70, 1.32] | 0.81 [0.59, 1.13] | **1.37 [1.01, 1.86]^** | 1.25 [0.97, 1.61] |
| Water | 0.73 [0.51, 1.03] | 0.95 [0.64, 1.42] | 0.73 [0.47, 1.14] | 1.01 [0.67, 1.54] | 1.02 [0.71, 1.46] |
| BMI | 0.06 [-0.29, 0.40] | -0.03 [-0.36, 0.30] | -0.22 [-0.49, 0.05] | 0.18 [-0.17, 0.54] | 0.07 [-0.22, 0.36] |
| Underweight | 1.28 [0.86, 1.90] | 1.44 [0.89, 2.33] | 0.98 [0.62, 1.55] | 0.80 [0.51, 1.24] | 0.83 [0.58, 1.19] |
| Overweight | 0.73 [0.52, 1.02] | 1.20 [0.80, 1.79] | 1.08 [0.69, 1.69] | 1.21 [0.75, 1.94] | 1.04 [0.72, 1.50] |

Significant (*p* < .05) associations are indicated in **bold font**;

[a] "I make sure that my child eats enough healthy food products";

[b] "I get my child enthusiastic about healthy products, such as vegetables, fruit and wholegrain products";

[c] BMI, as well as mean healthy and unhealthy food intake (each ranging from 1–3), were treated as continuous variables. For these outcomes, B values were calculated. Individual food categories were treated as ordinal

variables with three levels (1–3), while underweight and overweight status were treated as binary outcomes (yes/no). For the ordinal and binary outcomes, odds ratios (ORs) were calculated from the estimated regression coefficients.

[d] All models were adjusted for the child's BMI z-scores, sex, age, living situation, the mother's educational level, age, BMI, parity, and recruitment group;

^ Indicates associations no longer significant after correcting for multiple testing using the Benjamini–Hochberg procedure (1);

Note: All food parenting practices were assessed on a 5-point scale (1–5), using items from the Child Feeding Questionnaire (CFQ) (2) or adapted from Gubbels et al. (2011) (3).

## References

1. Thissen D, Steinberg L, Kuang D. Quick and easy implementation of the Benjamini-Hochberg procedure for controlling the false positive rate in multiple comparisons. Journal of educational and behavioral statistics. 2002;27(1):77-83.

2. Birch LL, Fisher JO, Grimm-Thomas K, Markey CN, Sawyer R, Johnson SL. Confirmatory factor analysis of the Child Feeding Questionnaire: a measure of parental attitudes, beliefs and practices about child feeding and obesity proneness. Appetite. 2001;36(3):201-10.

3. Gubbels JS, Kremers SP, Stafleu A, de Vries SI, Goldbohm RA, Dagnelie PC, et al. Association between parenting practices and children's dietary intake, activity behavior and development of body mass index: the KOALA Birth Cohort Study. International Journal of Behavioral Nutrition and Physical Activity. 2011;8:1-13.
